# Supplementary material for: Role of pKA in Charge Regulation and Conformation of Various Peptide Sequences
Source: Polymers (Basel). 2021 Jan 9;13(2):214. doi: 10.3390/polym13020214 (PMC7827592; doi:10.3390/polym13020214)
Supplement: Supplementary file 1 [file polymers-13-00214-s001.pdf]

Supplementary Materials: Role of pK_A in charge regulation and conformation of various peptide sequences

Raju Lunkad, Anastasiia Murmiliuk, Zdeněk Tošner, Miroslav Štěpánek, Peter Košovan*

Contents

S1 Simulations **1**

S1.1 Determination and validation of parameters for the CG model 1

S1.2 Comparison between one-bead and two-bead CG models 4

S2 Experiments **7**

S2.1 Potentiometric titrations 7

S2.2 NMR spectra 8

References **12**

S1. Simulations

S1.1. Determination and validation of parameters for the CG model

In our previous publication[1], we determined and validated parameters of the CG model for Glutamic acid and Histidine from the $\mathrm{Glu_5-His_5}$ peptide, and for Aspartic acid and Lysine from the $\mathrm{Lys_5-Asp_5}$ peptide. In the current manuscript, we provide a similar validation of the CG model of Tyrosine, needed for the $\mathrm{Tyr_5-Lys_5}$ and $\mathrm{Tyr_5-His_5}$ peptides. To validate the model, we compared the all-atom (AA) simulations with CG simulation at pH $= 13$ because at this pH the Tyr groups are fully charged, while the base groups are uncharged. Under these conditions the averages obtained using the CG model should match the AA simulations of fully charged tetramer $\mathrm{Tyr_4}$.

In Fig.S1, we show the probability distributions of distances between the central beads of the CG model or CA atoms of the AA model ($r_{\mathrm{C-C}}$), between the central beads and the charged acid group ($r_{\mathrm{A-C}}$), between the nearest-neighbour acid groups ($r_{\mathrm{A-A}}$), and next-nearest neighbour acid groups ($r_{\mathrm{A-NA}}$) from AA and CG simulations. The average values of these distributions are listed in TableS1. First, we used AA simulations to calculate $r_{\mathrm{C-C}}$ distance distributions between CA atoms in the peptide backbones from the AA simulations, and distances of the charged groups and the CA atoms ($r_{\mathrm{A-C}}$). We used these values as inputs for the CG model. In CG simulation, we measured $r_{\mathrm{C-C}}$ distance between the C beads in the $\mathrm{Tyr_5-His_5}$ and $\mathrm{Tyr_5-Lys_5}$ peptides. The average value of $r_{\mathrm{C-C}}$ and $r_{\mathrm{A-C}}$ in AA and CG models agree within the statistical uncertainty of approx. 1% , and all distributions show a single peak in both AA and CG simulations. Next, we validated the CG model by comparing the distances between the nearest-neighbour and next-nearest-neighbour acid groups on the side-chains. We expect that these distances are crucial for the correct prediction of the ionization degree. The average values of the $r_{\mathrm{A-A}}$ distances in AA and CG simulations agree within the statistical uncertainty of approx. 5% and also the shapes of the distributions are similar. On the contrary, the average values of the $r_{\mathrm{A-NA}}$ distances in AA and CG simulations do not agree very well. Specifically, AA simulations suggest that $r_{\mathrm{A-NA}} \lesssim r_{\mathrm{A-A}}$ while from CG simulations we obtain $r_{\mathrm{A-NA}} > r_{\mathrm{A-A}}$. The statistical uncertainty of approx. $20-30\%$ indicates that there are significant fluctuations in the $r_{\mathrm{A-NA}}$ distances within $\mathrm{Tyr_4}$ tetramer, which is also reflected by rather broad distributions. They are presumably caused by cis-trans conformational transitions, hydrogen bonds, or other specific interactions, which were not explicitly included in the CG model. Therefore, the shapes of the AA distributions that are not fully reproduced by the CG simulation. Thus, we may expect that

	AA of Tyr_4	CG of $Tyr_5 - His_5$	CG of $Tyr_5 - Lys_5$
r_{C-C}	0.385 ± 0.005	0.388 ± 0.005	0.389 ± 0.005
r_{A-C}	0.648 ± 0.012	0.647 ± 0.006	0.647 ± 0.006
r_{A-A}	1.139 ± 0.255	1.062 ± 0.093	1.062 ± 0.093
r_{A-NA}	0.974 ± 0.364	1.236 ± 0.175	1.236 ± 0.171

Table S1. Average distances between CA atoms on neighbouring amino acids or C beads, r_{C-C}, between the CA atoms or C beads and the charged group on the amino acids or A beads, r_{A-C}, between charged group on neighbouring amino acids or A beads, r_{A-A} and between charged group on next-nearest-neighbour amino acids or A beads, r_{A-NA}, of Tyrosine amino acids from the AA and CG simulations. Average distances from the CG simulations are taken from $Tyr_5 - His_5$ and $Tyr_5 - Lys_5$ peptides pH = 13.

CG simulations of peptides which contain Tyrosine might not yield as quantitative agreement with experiments as other peptides investigated in our previous study.[1] To obtain quantitative predictions, it would be desirable to use an augmented representation of Tyr side chains, which would account for the effects discussed above. Even though these might have significant consequences for the specific case of Tyrosine, they should not affect the general conclusions of the current manuscript regarding the role of ΔpK_A, alternating vs. diblock sequence and chain length of the peptide.

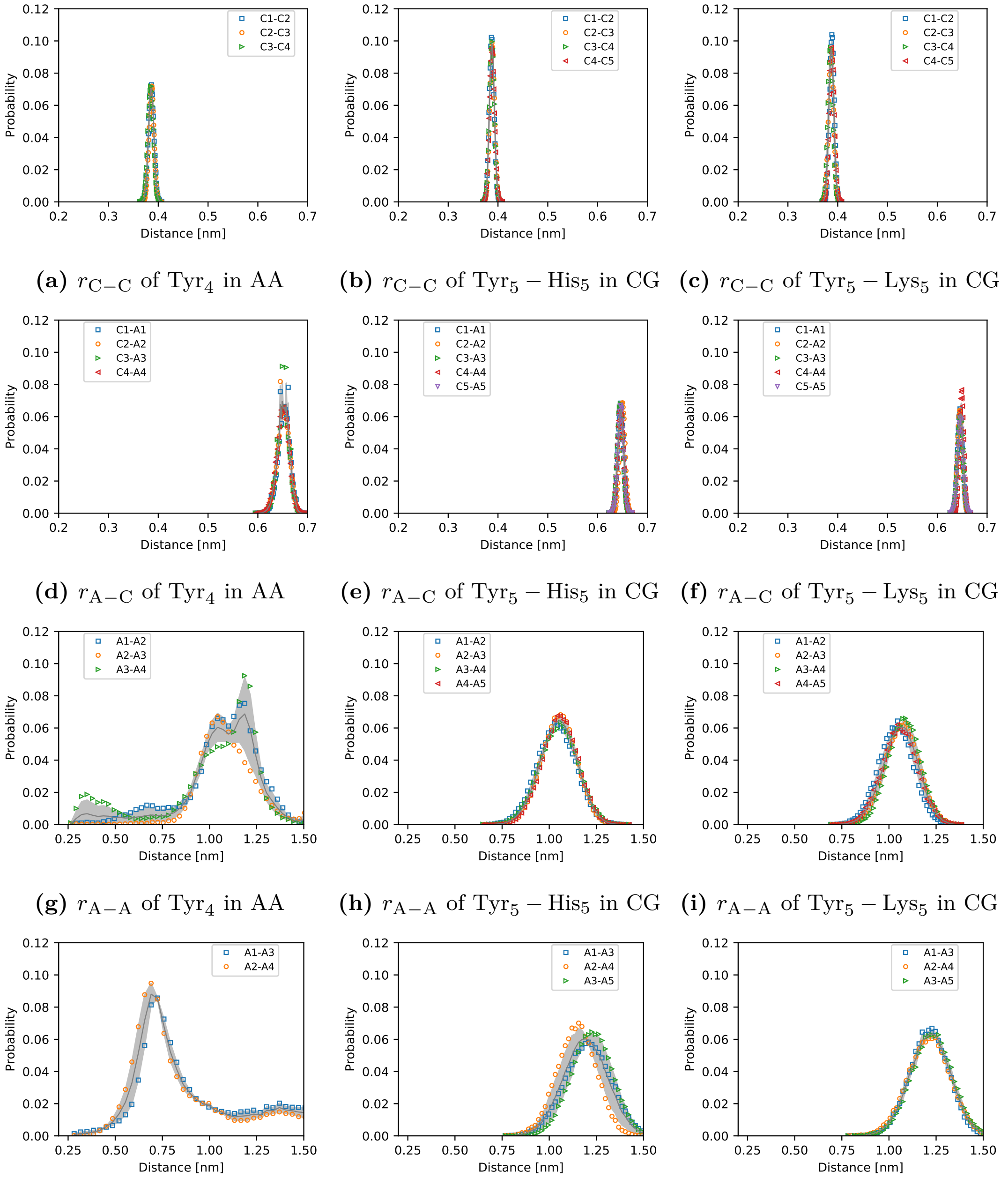

(a) $r_{\mathrm{C-C}}$ of Tyr_4 in AA **(b)** $r_{\mathrm{C-C}}$ of $\mathrm{Tyr}_5 - \mathrm{His}_5$ in CG **(c)** $r_{\mathrm{C-C}}$ of $\mathrm{Tyr}_5 - \mathrm{Lys}_5$ in CG

(d) $r_{\mathrm{A-C}}$ of Tyr_4 in AA **(e)** $r_{\mathrm{A-C}}$ of $\mathrm{Tyr}_5 - \mathrm{His}_5$ in CG **(f)** $r_{\mathrm{A-C}}$ of $\mathrm{Tyr}_5 - \mathrm{Lys}_5$ in CG

(g) $r_{\mathrm{A-A}}$ of Tyr_4 in AA **(h)** $r_{\mathrm{A-A}}$ of $\mathrm{Tyr}_5 - \mathrm{His}_5$ in CG **(i)** $r_{\mathrm{A-A}}$ of $\mathrm{Tyr}_5 - \mathrm{Lys}_5$ in CG

(j) $r_{\mathrm{A-NA}}$ of Tyr_4 in AA **(k)** $r_{\mathrm{A-NA}}$ of $\mathrm{Tyr}_5 - \mathrm{His}_5$ in CG **(l)** $r_{\mathrm{A-NA}}$ of $\mathrm{Tyr}_5 - \mathrm{Lys}_5$ in CG

Figure S1. Distribution of distances from the all-atom (AA) simulations of Tyrosine tetramers compared with the distributions from coarse-grained (CG) simulations of $\mathrm{Tyr}_5 - \mathrm{His}_5$ and $\mathrm{Tyr}_5 - \mathrm{Lys}_5$ peptides at $\mathrm{pH} = 13$. The symbol $r_{\mathrm{C-C}}$ refers to distance between C beads, $r_{\mathrm{A-C}}$ refers to distance between the C beads and A beads, $r_{\mathrm{A-A}}$ refers to distance between A beads on the nearest-neighbour amino acids, and $r_{\mathrm{A-NA}}$ refers to distance between A beads on the next-nearest-neighbour amino acids $r_{\mathrm{A-NA}}$ in the sequence. In the case of all-atom simulations, C beads correspond to CA atoms, and A beads correspond to the charged group on the acidic side-chain. Average values from these distributions are listed in Table S1.

S1.2. Comparison between one-bead and two-bead CG models

In the early stages of this work, we considered a simpler model of the peptides where each amino acid was represented by just one bead. In this one-bead model, the ionisable groups were located on the backbone, and the bond length between these beads was the only adjustable parameter. Below, we used the $Glu_5 - His_5$ peptide as an illustrative example to compare the results obtained using this one-bead CG model with two-bead CG model and with all-atom simulations. To compare the CG models with all-atom simulations of fully ionised tetramers, we used the CG simulation results at extreme pH values (1 and 13). Under these conditions, either the base or the acid block was fully ionised, and the CG results should match the AA simulations.

In the one-bead model, we chose the the bond length between the ionisable groups to match the distances between the charged groups on the nearest-neighbour side-chains measured from AA simulation [1]. Therefore, the average distances between the nearest-neighbour charges in the one-bead model almost perfectly reproduced the corresponding values from AA simulations, shown in Table S2. The two-bead model was constructed using the $r_{\mathrm{C-C}}$ and $r_{\mathrm{A-C}}$ distances as inputs rather than $r_{\mathrm{A-A}}$. Therefore, it did not reproduce the AA results for $r_{\mathrm{A-A}}$ distances as perfectly as the one-bead model. Nevertheless, the two-bead model still agrees with the AA results within the statistical uncertainty.

Similar to the nearest-neighbour distances, the two-bead CG model reasonably well reproduces also the average distances between the next-nearest neighbours, $r_{\mathrm{A-NA}}$, also shown in Table S2. In both cases (Glu and His), $r_{\mathrm{A-NA}}$ exceeds $r_{\mathrm{A-A}}$ by less than 20%, suggesting that the charged side-chains prefer trans conformations. On the contrary, the one-bead CG model yields $r_{\mathrm{A-NA}} \approx 1.5 r_{\mathrm{A-A}}$ because this model cannot discern the cis and trans conformations. The preference of trans conformations is further supported by the probability distributions of distances $r_{\mathrm{A-A}}$ and $r_{\mathrm{A-NA}}$, shown in Fig. S2 and S3. We observe that both CG models yield rather symmetric distributions that qualitatively resemble the Gaussian distribution. Expectedly, none of the CG models reproduced the fine details of distributions from AA simulations. Nevertheless, the most probable values of the distributions from two-bead CG model approximately coincide with the the AA model, and both types of distributions significantly overlap. On the contrary, the most probable values from the one-bead model are clearly shifted to higher values of $r_{\mathrm{A-NA}}$, and the distributions do not significantly overlap with those from the AA model. Very similar situation could be observed when comparing the average distances and their distributions within other model peptides simulated in this work and in our previous publication (data not shown).[1]

Finally, in Fig. S4 we compared the ionisation response of diblock peptides obtained from simulations using the one-bead and two-bead CG models. It turns out that both models provide very similar predictions but systematic differences can be found upon closer inspection. The two-bead model predicts a steeper change in the ionisation as a function of pH and its isoelectric point slightly differs from the ideal one because of the asymmetry in the interaction parameters of the acid and base groups. These differences proved significant when we were making quantitative comparisons with experiments in our previous publication.[1] It can be expected that the differences between one-bead and two-bead model would further diminish in other peptides or ampholytes, if the titratable groups were further from each other. In such ampholytes, the one-bead representation might provide equally good predictions as the two-bead representation.

	Glutamic groups		Histidine groups	
model type	$r_{\mathrm{A-A}}$	$r_{\mathrm{A-NA}}$	$r_{\mathrm{A-A}}$	$r_{\mathrm{A-NA}}$
all-atom (AA)	0.92 ± 0.16	1.03 ± 0.26	0.93 ± 0.18	1.10 ± 0.24
one-bead CG	0.92 ± 0.01	1.40 ± 0.11	0.93 ± 0.01	1.42 ± 0.11
two-bead CG	0.84 ± 0.11	1.03 ± 0.13	0.86 ± 0.12	1.02 ± 0.14

Table S2. Average distance between the charged groups on the nearest-neighbour amino acids, $r_{\mathrm{A-A}}$, and between next-nearest-neighbour amino acids, $r_{\mathrm{A-NA}}$, of Glutamic acid and Histidine within the $\mathrm{Glu_5 - His_5}$ peptide, obtained from the all-atom simulations and CG simulations using two different models. Average distances from the CG simulations between the Glutamic groups are taken from simulations of the $\mathrm{Glu_5 - His_5}$ peptide at pH $= 13$, whereas distances between the Histidine groups are taken from simulations at pH $= 1$.

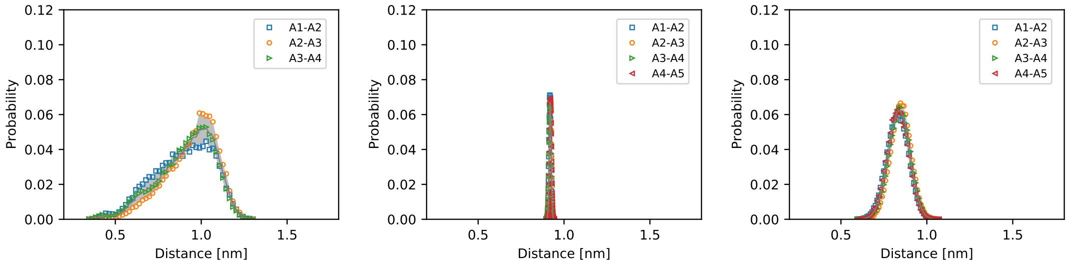

(a) $r_{\mathrm{A-A}}$ in AA **(b)** $r_{\mathrm{A-A}}$ in one-bead CG **(c)** $r_{\mathrm{A-A}}$ in two-bead CG

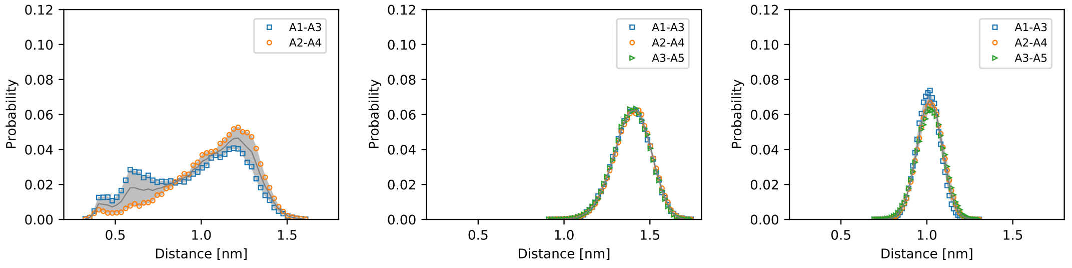

(d) $r_{\mathrm{A-NA}}$ in AA **(e)** $r_{\mathrm{A-NA}}$ in one-bead CG **(f)** $r_{\mathrm{A-NA}}$ in two-bead CG

Figure S2. Distribution of average distance between the charged groups on the nearest-neighbour side chains, $r_{\mathrm{A-A}}$, and next-nearest-neighbour side chains, $r_{\mathrm{A-NA}}$, from all-atom (AA) simulations of Glutamic acid tetramers compared with Glutamic gorups from coarse-grained (CG) simulations of $\mathrm{Glu_5 - His_5}$ peptide using one-bead and two-bead CG model at pH $= 13$. Average values from these distributions are listed in Table S2.

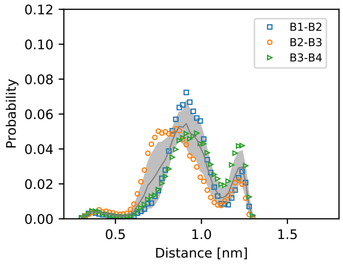

(a) $r_{\mathrm{A-A}}$ in AA

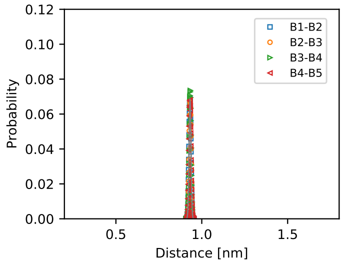

(b) $r_{\mathrm{A-A}}$ in one-bead CG

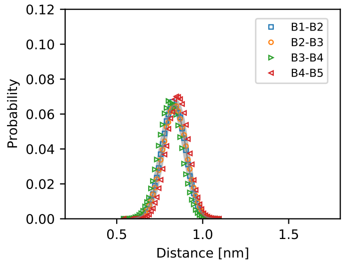

(c) $r_{\mathrm{A-A}}$ in two-bead CG

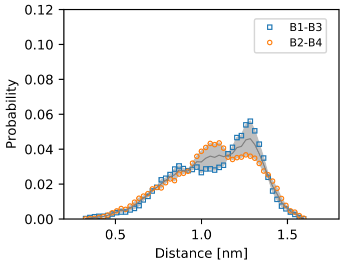

(d) $r_{\mathrm{A-NA}}$ in AA

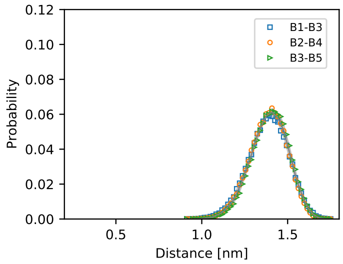

(e) $r_{\mathrm{A-NA}}$ in one-bead CG

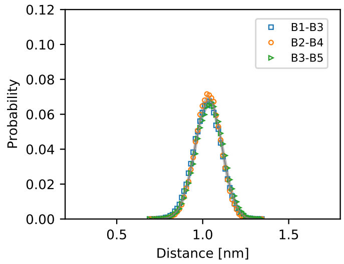

(f) $r_{\mathrm{A-NA}}$ in two-bead CG

Figure S3. Distribution of average distance between the charged groups on the nearest-neighbour amino acid side chain, $r_{\mathrm{A-A}}$, and next-nearest-neighbour side chains, $r_{\mathrm{A-NA}}$, from all-atom (AA) simulations of Histidine tetramers compared with Histidine gorups from coarse-grained (CG) simulations of $\mathrm{Glu}_5 - \mathrm{His}_5$ peptide using one-bead and two-bead CG model at pH = 1. Average values from these distributions are listed in Table S2.

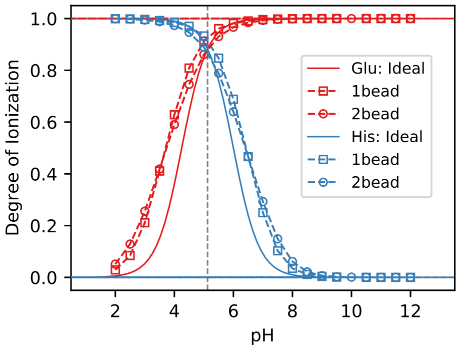

(a) $\mathrm{Glu}_5 - \mathrm{His}_5$

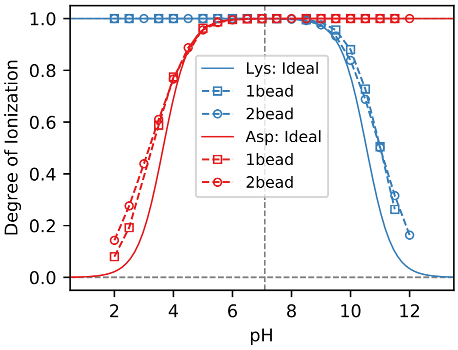

(b) $\mathrm{Lys}_5 - \mathrm{Asp}_5$

Figure S4. Comparison of the degree of ionisation as a function of pH obtained from the one-bead CG model, two-bead CG model and from the ideal Henderson-Hasselbalch equation.

S2. Experiments

S2.1. Potentiometric titrations

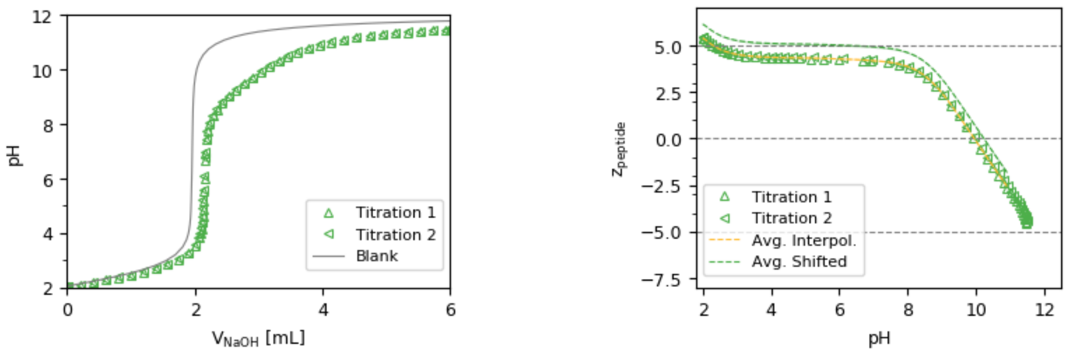

(a) Solution pH as a function of added volume of NaOH, V_{NaOH}.

(b) Charge on the peptide calculated from the measured pH and V_{NaOH}. The orange dashed line shows average of the two independent runs. The green dashed line shows the average shifted as described in the text.

Figure S5. Potentiometric titration of the peptides.

In Fig.S5a we show the potentiometric titration of $\mathrm{Tyr}_5 - \mathrm{Lys}_5$ from two repeated runs. Clearly, the raw data deviates from the ideal titration curves. In Fig.S5b we show the charge on the peptide calculated from the data in Fig.S5a. The obtained $z_{\mathrm{peptide}}(\mathrm{pH})$ curves from the two repeated runs were first linearly interpolated, then the interpolated curves were averaged, and finally the averaged curve was shifted to match $z_{\mathrm{peptide}} = +5$ at $\mathrm{pH} = 5$. By shifting the curve we accounted for the unknown amount of TFA counterions in the peptide sample, as discussed in detail in Ref.[1] The value of $\mathrm{pH} = 5$ was chosen arbitrarily so that it is approximately in the middle of the plateau region within which it is safe to assume that the base groups are fully ionized while the acid groups are non-ionized, yielding $z_{\mathrm{peptide}}(\mathrm{pH}) = +5$.

S2.2. NMR spectra

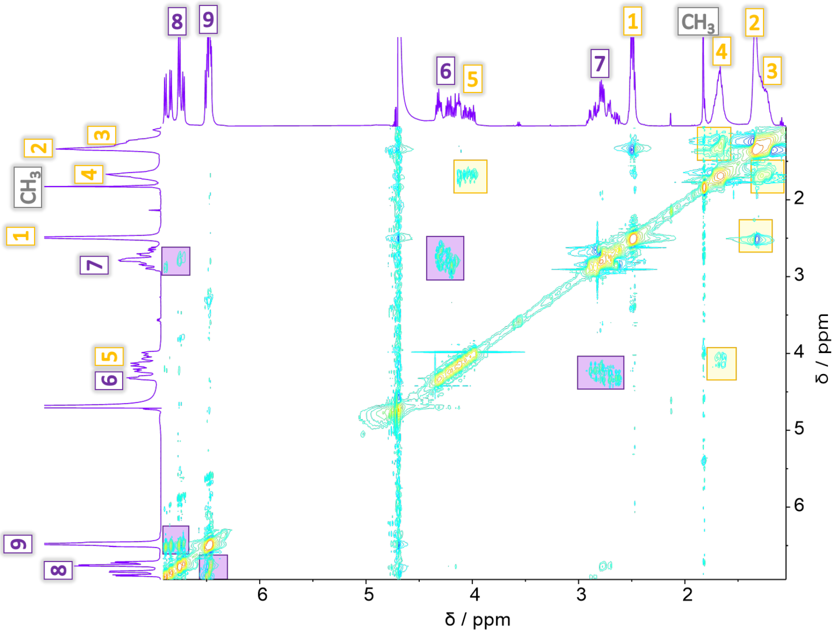

Figure S6. NOESY spectrum of $Tyr_5 - Lys_5$.

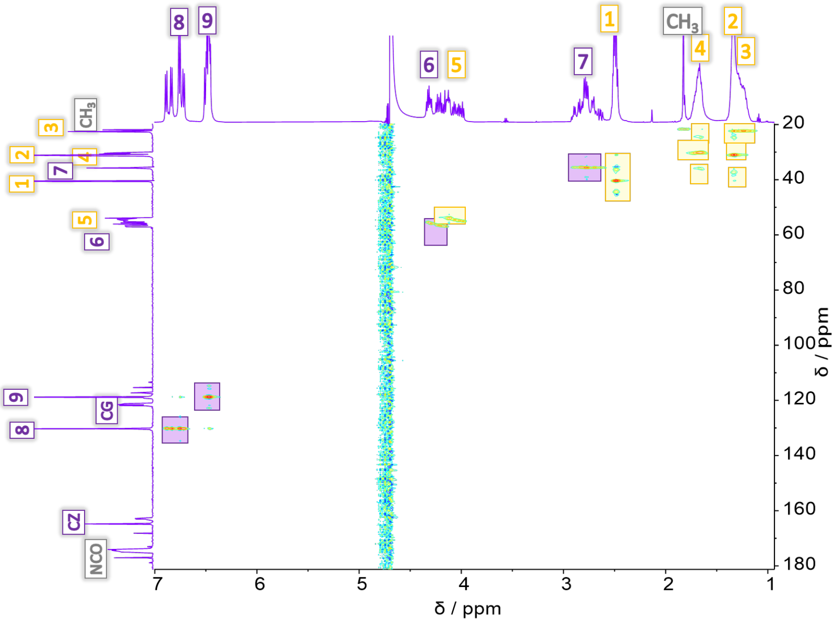

Figure S7. HSQC spectrum of $Tyr_5 - Lys_5$.

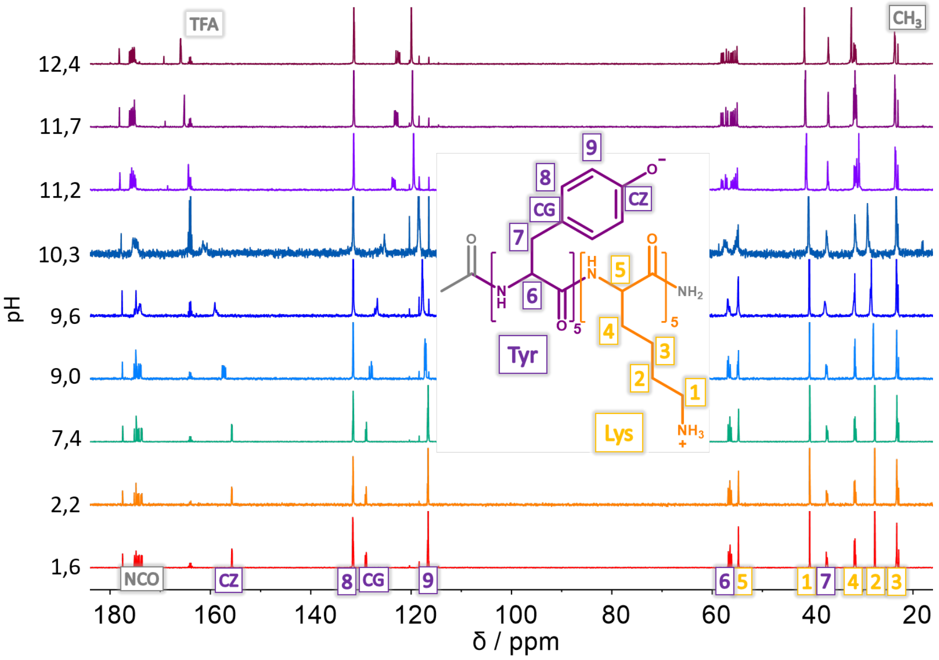

(a) 13 C spectrum

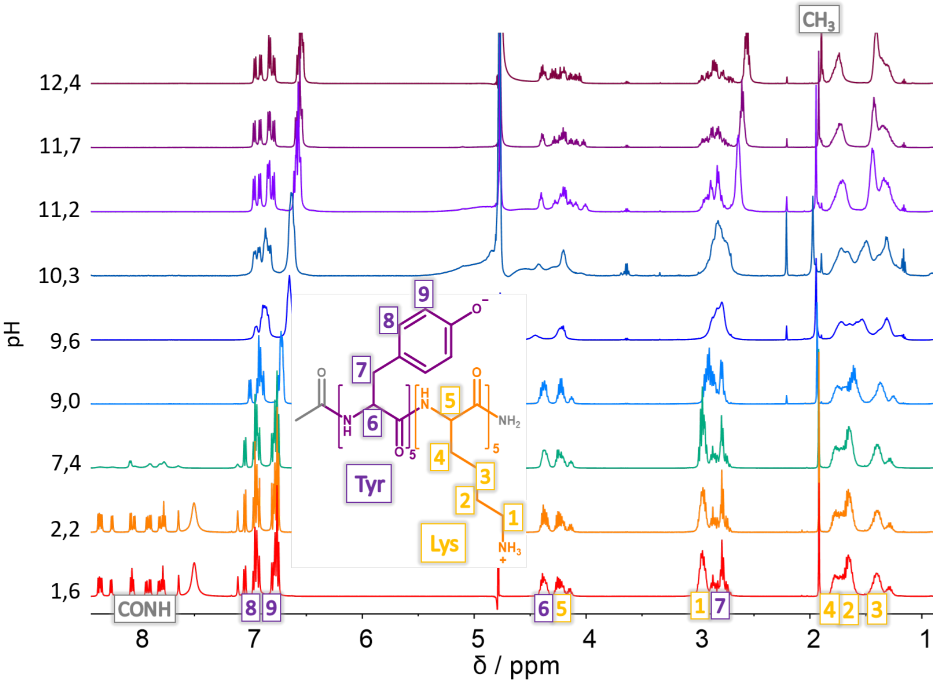

(b) 1 H spectrum

Figure S8. NMR spectra of $Tyr_5 - Lys_5$.

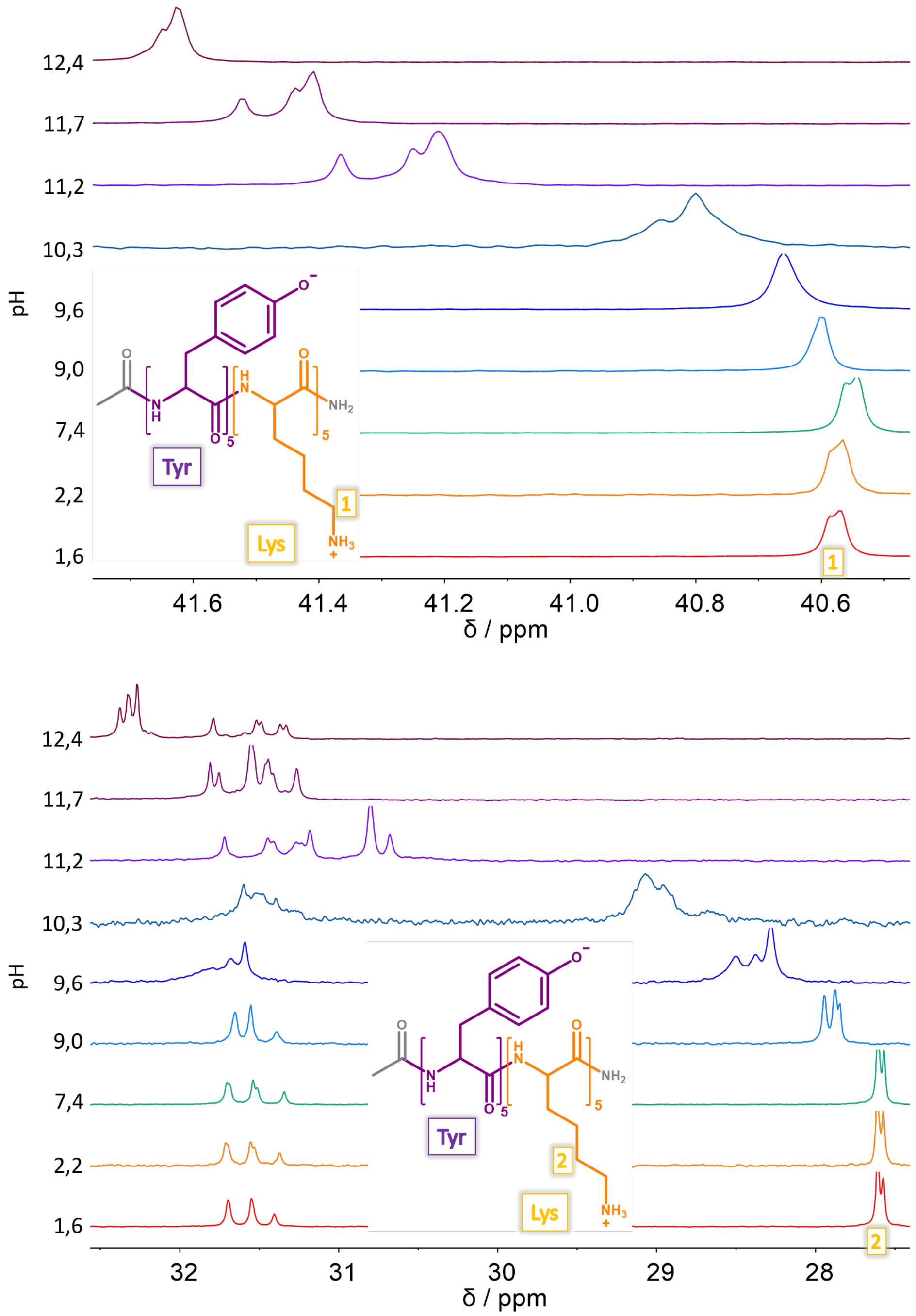

Figure S9. Details of NMR spectra of $Tyr_5 - Lys_5$, showing the peaks which we used to determine the degree of ionization of Lysine.

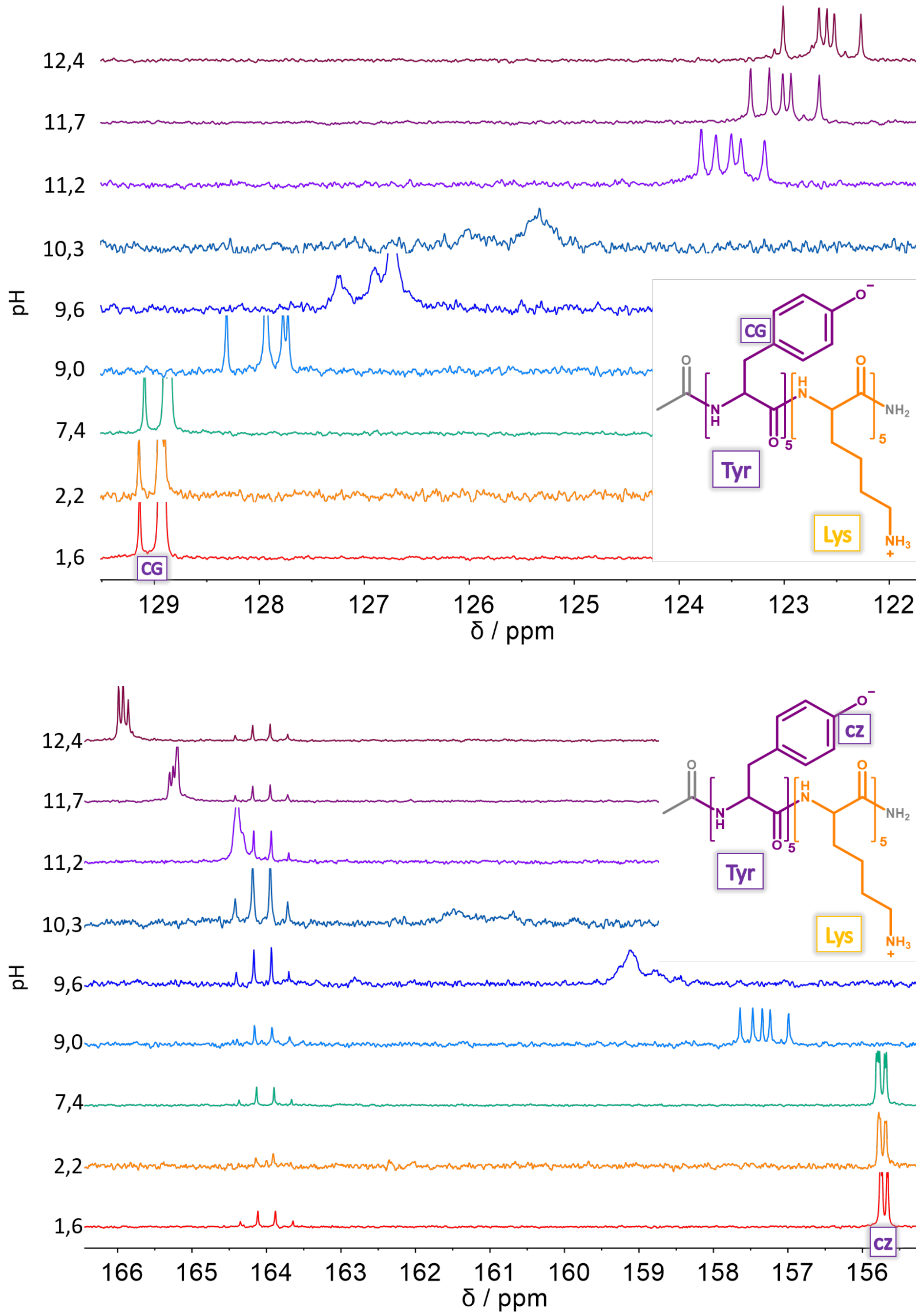

Figure S10. Details of NMR spectra of the $Tyr_5 - Lys_5$ peptide, showing the peaks which we used to determine the degree of ionization of Tyrosine.

References

1. Lunkad, R.; Murmiliuk, A.; Hebbeker, P.; Boublík, M.; Tošner, Z.; Štěpánek, M.; Košovan, P. Quantitative prediction of charge regulation in oligopeptides. *Molecular Systems Design & Engineering* **2021**. doi:10.1039/d0me00147c.
